# Identifying classes of the pain, fatigue, and depression symptom cluster in long-term prostate cancer survivors—results from the multi-regional Prostate Cancer Survivorship Study in Switzerland (PROCAS)

**DOI:** 10.1007/s00520-021-06132-w

**Published:** 2021-04-13

**Authors:** Salome Adam, Melissa S. Y. Thong, Eva Martin-Diener, Bertrand Camey, Céline Egger Hayoz, Isabelle Konzelmann, Seyed Mohsen Mousavi, Christian Herrmann, Sabine Rohrmann, Miriam Wanner, Katharina Staehelin, Räto T. Strebel, Marco Randazzo, Hubert John, Hans-Peter Schmid, Anita Feller, Volker Arndt

**Affiliations:** 1 National Institute for Cancer Epidemiology and Registration (NICER), c/o University of Zurich, Zurich, Switzerland; 2 Division of Chronic Disease Epidemiology, Epidemiology, Biostatistics and Prevention Institute, University of Zurich, Zurich, Switzerland; 3 Unit of Cancer Survivorship, German Cancer Research Center (DKFZ), Heidelberg, Germany; 4 Fribourg Cancer Registry, Fribourg, Switzerland; 5 Health Observatory Valais, Valais Cancer Registry, Sion, Switzerland; 6 Cancer Registry East Switzerland, St. Gallen, Switzerland; 7 Cancer Registry Graubünden and Glarus, Chur, Switzerland; 8 Cancer Registry Zurich, Zug, Schaffhausen and Schwyz, University Hospital Zurich, Zurich, Switzerland; 9 Basel Cancer Registry, Cantonal Department of Health, Basel, Switzerland; 10 Department of Urology, Graubünden Cantonal Hospital, Chur, Switzerland; 11 Department of Urology, GZO Spital Wetzikon AG, Wetzikon, Switzerland; 12 Department of Urology, Winterthur Cantonal Hospital, Winterthur, Switzerland; 13 Department of Urology, School of Medicine (Med-HSG), St. Gallen, Switzerland

**Keywords:** Prostate cancer, Classes, Pain, Fatigue, Depression, Symptom cluster

## Abstract

**Purpose:**

Aside from urological and sexual problems, long-term (≥5 years after initial diagnosis) prostate cancer (PC) survivors might suffer from pain, fatigue, and depression. These concurrent symptoms can form a cluster. In this study, we aimed to investigate classes of this symptom cluster in long-term PC survivors, to classify PC survivors accordingly, and to explore associations between classes of this cluster and health-related quality of life (HRQoL).

**Methods:**

Six hundred fifty-three stage T1-T3N0M0 survivors were identified from the *Prostate Cancer Survivorship in Switzerland* (PROCAS) study. Fatigue was assessed with the EORTC QLQ-FA12, depressive symptoms with the MHI-5, and pain with the EORTC QLQ-C30 questionnaire. Latent class analysis was used to derive cluster classes. Factors associated with the derived classes were determined using multinomial logistic regression analysis.

**Results:**

Three classes were identified: class 1 (61.4%) – “low pain, low physical and emotional fatigue, moderate depressive symptoms”; class 2 (15.1%) – “low physical fatigue and pain, moderate emotional fatigue, high depressive symptoms”; class 3 (23.5%) – high scores for all symptoms. Survivors in classes 2 and 3 were more likely to be physically inactive, report a history of depression or some other specific comorbidity, be treated with radiation therapy, and have worse HRQoL outcomes compared to class 1.

**Conclusion:**

Three distinct classes of the pain, fatigue, and depression cluster were identified, which are associated with treatment, comorbidities, lifestyle factors, and HRQoL outcomes. Improving classification of PC survivors according to severity of multiple symptoms could assist in developing interventions tailored to survivors’ needs.

**Supplementary Information:**

The online version contains supplementary material available at 10.1007/s00520-021-06132-w.

## Introduction

Cancer survivors often suffer from multiple symptoms, depending on their cancer and therapy [1–3]. Numerous studies have already shown that symptoms or the experienced symptom burden impact cancer survivors’ health-related quality of life (HRQoL) and clinical outcomes [4]. However, symptom management studies have traditionally focussed only on single symptoms [5], although research indicates that multiple symptoms often coexist and could form symptom clusters [6, 7]. These symptom clusters can be of therapeutic importance. Treating one symptom of the cluster could influence the others, as the direct treatment of one symptom may indirectly have an impact on another symptom in the cluster [6, 8]. Consequently, treating one symptom may not necessarily improve HRQoL or prognosis. Therefore, a more profound understanding of symptom clusters and how they affect cancer survivors is necessary.

A symptom cluster has been defined as a stable group of two or more concurrent symptoms that are related and distinct from other symptom clusters [7]. Symptom cluster composition can differ by age, sex, performance status, and cancer diagnosis [9, 10]. Moreover, the presence of specific clusters and the number and severity of symptoms are associated with survival/mortality and poorer HRQoL [9, 11–14]. However, most pertinent studies so far relied on data of breast and lung cancer patients [15], whereas information on symptom clusters in other cancer types, for example prostate cancer (PC), is rare.

Long-term (cancer patients surviving the initial diagnosis for ≥5 years [16]) PC survivors may often suffer from pain, fatigue, and depression in addition to common urological and sexual problems [17–19]. Up to 40% of a heterogeneous group of PC survivors reported to be chronically fatigue after various treatments [20], up to 50% suffered from chronic pain [21], and post-treatment depression prevalence can be up to 18.5% [22]. Pain, fatigue, and depression frequently co-occur, and could therefore be considered a symptom cluster [6, 23]. Prevalence of this pain-fatigue-depression cluster ranges from 7% in survivors of PC [23] to 21.4 % in patients with advanced cancers of the lung or pancreas [6]. However, these studies neither identified classes of this cluster nor categorized survivors into the identified classes. Even though identifying classes of a symptom cluster and better classification of survivors to the identified classes are important to understand which survivor needs more intensive symptom management [24]. Research investigating the cluster of cognitive disturbance, sleep problems, pain, depression, and fatigue, referred to as the psychoneurological symptom cluster [25] found four distinct subgroups: (1) all low symptoms, (2) high fatigue and low pain, (3) high pain, and (4) all high symptoms [24, 26]. Patients in these subgroups differed with regard to clinical and demographic characteristics. Moreover, the subgroup with low levels of all four symptoms reported the highest HRQoL [24]. However, to our knowledge, no published study has identified classes of the pain-fatigue-depression cluster in PC survivors, even though it is a relatively common symptom cluster [23]. Therefore, in this exploratory analysis, our first objective was to identify possible classes of the pain-fatigue-depression symptom cluster in a large population-based sample of long-term stage T1-T3N0M0 PC survivors. Our second objective was to identify factors associated with the derived classes and explore associations between classes of the cluster and HRQoL.

## Methods

### Study design and study population

Participants were included from the multi-regional *Prostate Cancer Survivorship in Switzerland* (PROCAS) cohort. Details of the PROCAS study recruitment and data collection design have been described elsewhere [27]. In short, the PROCAS study included 748 long-term (cancer patients surviving the initial diagnosis for ≥5 years) PC survivors younger than 75 years of age at diagnosis and diagnosed between 2006 and 2011. They were identified via six population-based cancer registries (Cancer Registry Fribourg, Cancer Registry Basel, Cancer Registry Graubünden and Glarus, Cancer Registry East Switzerland, Valais Cancer Registry, Cancer Registry Zurich and Zug) covering an underlying population of over 3.4 million inhabitants (~40% of the total population in Switzerland) in both German- and French-speaking Switzerland. The identified PC patients were invited to participate in the study by their treating urologists. Questionnaires and all other study documents were available in German, French, and Italian. Data collection was conducted between 2017 and 2018 by postal questionnaire. Non-respondents received one reminder. Of the 8712 survivors who met the inclusion criteria for the study (Figure S1), 1246 were randomly selected for participation, of whom 1194 could be contacted and received an invitation. Finally, 748 returned a completed questionnaire (response rate: 62.2%). This analysis was restricted to 653 PC survivors staged T1–T3 N0 and M0 (according to the TNM classification system published by the American Joint Committee on Cancer [28]).

### Study measurements

#### Fatigue

The EORTC QLQ-FA12 is a fatigue module developed to complement the European Organization for Research and Treatment of Cancer Quality of Life Core Questionnaire (EORTC QLQ-C30) [29]. The questionnaire consists of ten unidirectional items and two criteria variables. Responses are arranged on a 4-point scale (1: “not at all” to 4: “very much”). Two criteria variables measure the extent to which fatigue interferes with daily activities and social life. Ten items are assigned to three subscales: physical, emotional, and cognitive fatigue. According to EORTC scoring procedures, all scores are standardized to a range of 0 to 100 [30]. Higher scores indicate higher fatigue.

#### Pain

Pain was assessed using the pain subscale of the EORTC QLQ-C30. The scale consists of two questions. According to EORTC scoring procedures, all scores are standardized to a range of 0 to 100 [30]. Higher score indicates more pain.

#### Depressive symptoms

Depressive symptoms were measured with the Mental Health Inventory (MHI)-5, which is a five-item mental health measure of SF-36 [31]. Responses are arranged on a 5-point scale (1: “always” to 5: “never”). The scores were standardized by linear transformation to a scale ranging from 0 to 100 with higher scores indicating lower level of depressive symptoms. We defined depressive symptoms using a cut-off of ≤56 [32].

#### HRQoL and PC-specific symptom burden

We used the five functioning (physical, role, emotional, cognitive, social) and the health status/overall quality of life scales of the EORTC QLQ-C30 questionnaire to assess HRQoL and the PC-specific module QLQ-PR25 to assess the PC-specific symptom burden. The PC-specific EORTC QLQ-PR25 questionnaire includes 25 questions, assessing urinary and bowel symptoms, sexual activity, sexual functioning, and hormonal treatment–related symptoms. Items for the EORTC QLQ-C30 functioning and EORTC QLQ-PR25 subscales were scored on a scale from 1 (not at all) to 4 (very much), and from 1 (very poor) to 7 (excellent) for items in the health status/overall quality of life scale. Scoring of all instruments was performed according to pertinent scoring manuals [30, 33] and scores were linearly transformed to a scale of 0–100. Higher scores on the functioning scales and global health/QoL indicate better functioning and better health. Higher scores in the EORTC QLQ-PR25 represent a greater symptom burden or a better sexual functioning and more sexual activity.

#### Demographics, lifestyle, and clinical data

Cancer registries provided demographic parameters and clinical information such as date of birth, date of diagnosis, and cancer stage. Physicians and cancer registries gave detailed information on treatments, disease progression/relapse (including biochemical and clinical recurrence, and metastasis after diagnosis of primary tumour at time of survey), and other primary tumours. Self-reported information included education, living with partner, nationality, working status, body weight, body height, and physical activity. Furthermore, self-reported experience (yes/no) of the following comorbidities were assessed: depression, arthritis/rheumatism/arthrosis, diabetes, degenerative disc disease, and upper gastrointestinal disease.

### Statistics

For descriptive purposes, we compared clinical and sociodemographic characteristics between respondents and non-respondents using parametric tests. Non-parametric tests were applied when normality and homogeneity assumptions were violated.

As there are no established cut-offs in guidelines regarding the EORTC QLQ-C30 and EORTC QLQ-FA12 questionnaires, we dichotomized the scales of pain and emotional and physical fatigue using the 75th percentile as cut-off (Table 1) to identify PC survivors who suffered from fatigue or pain. There is a precedence to dichotomize these scales in order to facilitate the clinical utility of such scores [34]. For mental distress, the established cut-off ≤56 was used [32]. We excluded cognitive fatigue because the scores were very low (skewed to the left), suggesting that PC survivors in our sample do not have complaints on this aspect of fatigue. Deriving descriptions of the identified classes was performed based on visual comparison, as it was done in comparable papers [24, 26, 35]. Correlations were calculated to assess whether items were interrelated with HRQoL scales.

**Table 1:** Distribution of scores for the fatigue (physical, emotional), pain, and depression symptom cluster of respondents (*n* = 653)

	Missing values (*n*)	Mean score	SE	75th interquartile score
Physical fatigue	5	22.3	0.9	33.3
Emotional fatigue	8	10.9	1.2	11.1
Pain	1	15.8	0.9	33.3
Depressive symptoms*	3	70.3	0.6	80.0

Higher scores mean higher symptom burden

*For depressive symptoms, the reported cut-off of 56 was used

We performed latent class analysis (LCA) to identify groups of PC survivors with similar profiles of the pain-fatigue-depression symptom cluster. LCA is a probabilistic clustering approach that aims to obtain the smallest number of groups with similar profiles based on a categorical latent variable [36]. We used the four dichotomized scores of pain, depressive symptoms, and emotional and physical fatigue. The optimal number of latent classes was based on the model with the lowest Bayesian information criterion (BIC) value, indicating the best fit. Respondents were assigned to the class for which the posterior probability was highest.

Multinomial logistic regression was used to identify factors that discriminate between the identified classes. For these models, predictor variables were dichotomized [37]. As we were interested in the independent effect of each variable, we did not adjust for possible confounding in the multinomial logistic regression models in general. However, as age is a strong confounder and could be associated with each independent variable that we tested, sub-analyses were performed for all models adjusting for age (data not shown).

Multivariable linear models were calculated to describe and test for differences in HRQoL by the identified classes. These models were adjusted for cancer stage, age at survey, time since diagnosis, and external-beam radiation therapy. Other variables such as androgen deprivation therapy and radical prostatectomy were considered additional potential confounders but were not included in the final models as they did not improve the model fit (maximum likelihood test with *p* < 0.1). Independent variables were checked for multicollinearity by calculating the variance inflation factors (VIF) in all models. The *p*-values were not adjusted for multiple testing and refer to the individual tests rather than a global test for differences. All analyses were performed using STATA statistical software (version 15.1).

### Ethics

The PROCAS study was approved as a multi-centre study by the Ethics Committee Zurich and by all reviewer boards accountable for the participating cancer registries (BASEC Number: 2016-00608).

## Results

Mean age at survey was 72.9 (SD = 6.3) years and mean time since diagnosis 7.6 years (SD = 1.5) (Table 2). A majority of participants were Swiss, living with their partner, and had cancer stage T2N0M0. Respondents were statistically significantly younger (*p* = 0.023) than non-respondents, as well as more likely to be Swiss (*p* = 0.001), and to live with their partner (*p* = 0.045). Most participants were treated with radical prostatectomy (76.7%), followed by external-beam radiation therapy (29.6%).

**Table 2:** Characteristics of respondents and non-respondents

	Respondents	Non-respondents	*p*-value
	*n* = 653	*n* = 383	
	Col%	Col%	
Age at survey			
70 years	27.9	23.8	
70–74 years	31.4	26.9	
75–79 years	24.7	31.3	
≥80 years	16.1	18.0	**0.029**
Mean (SD)	72.9 (6.3)	73.8 (6.3)	**0.023**
Nationality Swiss (yes)	90.8	80.7	
No	4.1	12.8	
Unknown	5.1	6.5	**0.001**
Living with partner (yes)	70.6	62.6	
No	12.7	18.0	
Unknown	16.7	19.4	**0.045**
Histological grade			
1	0.5	1.3	
2	46.1	50.4	
3	31.7	29.8	
Unknown	21.8	18.5	0.1003
Cancer stage			
T1N0M0	16.9	25.1	
T2N0M0	64.8	54.3	
T3N0M0	19.3	20.6	0.0924
Years since diagnosis			
5–6	26	26.9	
7–8	44.1	29.7	
9–10	29.9	33.4	0.537
Mean (SD)	7.6 (1.5)	7.7 (1.5)	0.537
Therapy			
Radical prostatectomy	76.7	-	
External-beam radiation therapy	29.6	-	
Brachytherapy	5.8	-	
Androgen deprivation therapy	17.1	-	-

*Col* column

Correlations between the pain-fatigue-depression symptom cluster with EORTC QLQ-C30 and EORTC QLQ-PR25 scores were weak to moderate (correlation coefficient, − 0.50 to 0.40) (Table S2).

### Characteristics of identified PNS classes

Of the four classes identified with LCA, we selected a 3-class solution based on the lowest BIC (Table 3). Ten cases with missing data on at least one of the scales were excluded. Most PC survivors (*n* = 394, 61.4%) were categorized into class 1, 98 (15.1%) to class 2, and 151 (23.5%) to class 3.

**Table 3:** Optimal number of classes according to Bayesian Information Criterion (BIC)

	BIC	*p*-value	Entropy
2-cluster	2640	<0.001	0.8190
3-cluster	2631	0.010	0.6784
4-cluster	2633	1.000	0.6222

**Fig. 1:**
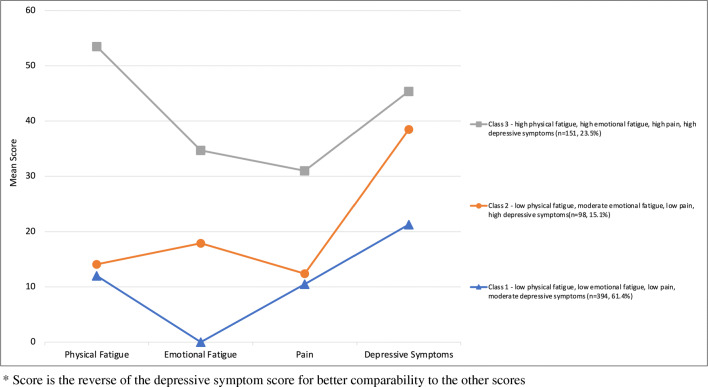
Mean scores of physical fatigue, emotional fatigue, pain, and depressive symptoms*, by class of pain-fatigue-depression cluster.

Class 1 is characterized by low scores for pain (mean = 10.5), and physical and emotional fatigue, and moderate scores for depressive symptoms (Fig. 1, Table S3). Low physical fatigue and pain scores but moderate emotional fatigue and high depressive symptoms scores characterized class 2. Class 3 was defined by high scores for all symptoms.

### Factors associated with identified pain-fatigue-depression symptom cluster classes

Multinomial logistic regression revealed that pain-fatigue-depression symptom cluster classes 1 and 2 differed significantly by having reported a depression as comorbidity (Table 4). In comparison with class 1 (low pain, low physical and emotional fatigue, moderate depressive symptoms), PC survivors in class 2 (low physical fatigue, low pain, moderate emotional fatigue, high depressive symptoms) were 9.5 times (95%CI: 3.94–23.01) more likely to have reported a depression.

**Table 4:** Odd ratios and 95%CIs of factors associated with latent classes of pain-fatigue-depression cluster

	Class 2 vs. class 1*		Class 3 vs. class 1*		Class 3 vs. class 2*	
	OR	95%CI	OR	95%CI	OR	95%CI
Age (years) at time of survey^1^						
<73	1		1		1	
≥73	0.81	0.51–1.31	**1.53**	**1.05**–**2.26**	1.69	0.96–3.00
Education (highest degree)						
Low and medium	1		1		1	
High	0.92	0.59–1.44	0.92	0.51–1.09	0.81	0.48–1.36
Nationality Swiss						
No	1		1		1	
Yes	1.41	0.40–4.91	0.57	0.26–1.25	0.41	0.11–1.50
Having a partner						
No	1		1		1	
Yes	1.03	0.48–2.20	0.53	**0.31**–**0.91**	0.52	0.23–1.16
Working at survey						
No	1		1		1	
Yes	0.85	0.41–1.75	0.82	0.44–1.52	0.97	0.42–2.25
Body mass index						
<25	1		1		1	
≥25	0.85	0.54–1.34	**2.23**	**1.44**–**3.45**	**2.62**	**1.50**–**4.59**
Vigorous physical activities (hours per week)^2^						
<1.25	1		1		1	
≥1.25	0.97	0.61–1.53	**0.45**	**0.30**–**0.67**	**0.46**	**0.27**–**0.79**
Light physical activities (hours per week)^1^						
<6	1		1		1	
≥6	0.66	0.42–1.04	0.69	0.47–1.00	1.03	0.62–1.72
Cancer stage						
T1–T2N0M0	1					
T3N0M0	0.76	0.41–1.41	1.20	0.96–1.51	1.92	0.98–3.79
Years since diagnosis						
5–7 years						
8–10 years	0.81	0.52–1.26	1.00	0.82–1.20	1.22	0.73–2.04
Disease progression/relapse						
No	1		1		1	
Yes	1.40	0.83–2.37	1.09	0.68–1.75	0.78	0.43–1.44
Most common comorbidities						
Arthritis/rheumatism/arthroses						
No	1		1		1	
Yes	1.22	0.72–2.08	1.81	1.18 – 2.80	1.48	0.82–2.68
Degenerative disc disease						
No						
Yes	1.25	0.67–2.33	**2.35**	**1.46**–**3.80**	1.89	0.97–3.68
Upper gastrointestinal disease						
No	1		1		1	
Yes	1.82	0.91–3.63	**2.11**	**1.18**–**3.89**	1.61	0.55–2.44
Diabetes						
No	1		1		1	
Yes	1.07	0.48–2.41	1.78	0.97–3.26	1.66	0.69–3.96
Depression						
No	1		1		1	
Yes	**9.52**	**3.94**–**23.01**	**15.97**	**7.19**–**35.50**	1.67	0.87–3.25
Therapy						
Radical prostatectomy						
No	1		1		1	
Yes	1.45	0.79–2.65	0.70	0.45–10.80	**0.48**	**0.25–0.92**
External-beam radiation therapy						
No	1		1		1	
Yes	0.73	0.43–1.24	**1.69**	**1.14**–**2.51**	**2.32**	**1.30**–**4.17**
Brachytherapy						
No	1		1		1	
Yes	2.01	0.88–4.59	1.24	0.44–2.80	0.62	0.24–1.63
Androgen deprivation therapy						
No	1		1		1	
Yes	1.03	0.56–1.91	1.56	0.97–2.49	1.51	0.77–3.00

Odds ratios based on two multinomial logistic regression models, whereas in the first model class 1 was the reference and in a second model class 2 was the reference

*Indicates reference group^1^ cut-off based on median^2^ cut-off based on recommendation of doing at least 1.25 h/week of vigorous-intensity sport activity

Class 1 - low physical fatigue, low emotional fatigue, low pain, moderate depressive symptoms (*n* = 364, 61.4%)

Class 2 - low physical fatigue, moderate emotional fatigue, low pain, high depressive symptoms (*n* = 98, 51.1%)

Class 3 - high physical fatigue, high emotional fatigue, high pain, high depressive symptoms (*n* = 151, 23.5%)

Missing values are below <5%

When compared to class 1, PC survivors in class 3 (high physical and emotional fatigue, high pain, high depressive symptoms) were more likely to be older (OR = 1.53, 95%CI: 1.05–2.26), to be overweight (OR = 2.23, 95%CI: 1.44–3.45), to have degenerative disc disease (OR = 2.35, 95%CI: 1.46–3.80), to have an upper gastrointestinal disease (OR = 2.11, 95%CI: 1.18–3.89), to have depression (OR = 15.97, 95%CI: 7.19–35.50), and to be treated with external-beam radiation therapy (OR = 1.69, 95%CI: 1.14–2.51). On the other hand, they were less likely to have a partner (OR = 0.53, 95%CI: 0.31–0.91), to do the recommended ≥1.25 h of vigorous physical activity per week (OR = 0.45, 95%CI: 0.30–0.67), and to have arthritis/rheumatism/arthrosis (OR = 1.81, 95%CI: 1.18–2.80).

When comparing class 2 (reference) and class 3, being overweight (OR = 2.62, 95%CI: 1.50–4.59), doing less vigorous physical activity per week (OR = 0.46, 95%CI: 0.27–0.79), being less likely to be treated with radical prostatectomy (OR = 0.48, 95%CI: 0.25–0.92), but more likely to be treated with external-beam radiation therapy (OR = 2.32, 95%CI: 1.30–4.17) were associated with being in class 3.

In age-adjusted multinomial logistic regression models, similar effects for each independent variable were observed (data not shown).

### Differences in HRQoL and PC-specific symptom burden by pain-fatigue-depression symptom cluster classes

Beside sexual activity, PC survivors in class 1 reported statistically significantly better functioning scores, lower symptom scores, and better sexual functioning (mean difference = 7.2, *p* = 0.003), when compared to survivors of class 3 (Table 5). A similar picture was observed when survivors of class 3 were compared to those of class 2, except for urinary bother (mean difference = − 3.3, *p* = 0.437), sexual activity (mean difference = − 0.7, *p* = 0.672), and sexual functioning (mean difference = − 0.8, *p* = 0.981) where no difference was observed. In comparison to survivors of class 1, survivors of class 2 indicated statistically significantly lower global health (mean difference = 6.8, *p* < 0.001) and functioning scores but similar physical functioning (mean difference = − 0.1, *p* = 0.963). Regarding symptoms, survivors in class 2 reported higher burden for urinary symptoms (mean difference = − 4.4, *p* = 0.015), bowel symptoms (mean difference = − 2.9, *p* = 0.009), hormone treatment–related symptoms (mean difference = − 5.0, *p* < 0.001), and worse sexual functioning (mean difference = 8.0, *p* = 0.005). All VIF in these models were below 2.

**Table 5:** EORTC QLQ-C30 and PR-25 scores according to the classes of the pain-fatigue-depression symptom cluster

	Class 1		Class 2		Class 3		Difference Class 1–Class 2		Difference Class 1–Class 3		Difference Class 2–Class 3	
	Mean	SE	Mean	SE	Mean	SE	Mean	*p*-value	Mean	*p*-value	Mean	*p*-value
EORTC QLQ-C30 scales												
Global health/QoL	84.0	0.8	77.2	1.7	63.6	1.4	**6.8**	**<0.001**	**20.4**	**<0.001**	**13.6**	**<0.001**
Physical functioning	93.9	0.8	94.0	1.4	77.9	1.1	− 0.1	0.963	**16**	**<0.001**	**16.1**	**<0.001**
Cognitive functioning	93.6	1.0	88.1	2.1	63.9	1.7	**5.5**	**0.019**	**29.7**	**<0.001**	**24.2**	**<0.001**
Emotional functioning	93.7	0.8	81.2	1.5	65.9	1.2	**12.5**	**<0.001**	**27.8**	**<0.001**	**15.3**	**<0.001**
Role functioning	90.7	0.8	84.5	1.6	74.6	1.3	**6.2**	**0.001**	**16.1**	**<0.001**	**9.9**	**<0.001**
Social functioning	92.4	1.1	83.7	2.1	71.3	1.7	**8.7**	**<0.001**	**21.1**	**<0.001**	**12.4**	**<0.001**
EORTC QLQ-PR25 scales												
Urinary symptoms	14.4	0.8	18.8	1.6	27.4	1.2	**− 4.4**	**0.015**	**− 13**	**<0.001**	**− 8.6**	**<0.001**
Urinary bother^1^	23.1	3.3	29.5	5.3	32.8	4.4	− 6.4	0.381	− 9.7	0.037	− 3.3	0.437
Bowel symptoms	3.2	0.5	6.1	1.0	11.7	0.8	**− 2.9**	**0.009**	**− 8.5**	**<0.001**	**− 5.6**	**0.001**
Hormonal treatment-related symptoms	7.5	0.5	12.5	1.1	19.6	0.8	**− 5.0**	**<0.001**	**− 12.1**	**<0.001**	**− 7.1**	**<0.001**
Sexual activity	43.4	1.4	38.1	2.7	38.8	2.2	**5.3**	**0.043**	4.6	0.193	− 0.7	0.672
Sexual functioning^1^	49.0	1.3	41.0	2.7	41.8	2.3	**8.0**	**0.005**	**7.2**	**0.003**	− 0.8	0.981

EORTC QLQ-C30: higher scores on functioning scales indicate better functioning or global health

EORTC QLQ-PR25: higher score in the EORTC QLQ-PR25 represents a greater symptom burden or better sexual functioning and activity

^1^Smaller sample sizes and no imputation was performed as the questions referring to these scales are conditional

Linear models were adjusted for cancer stage, age at survey, time since diagnosis, and external-beam radiation therapy

Class 1 - low physical fatigue, low emotional fatigue, low pain, moderate depressive symptoms (*n* = 364, 61.4%)

Class 2 - low physical fatigue, moderate emotional fatigue, low pain, high depressive symptoms (*n* = 98, 51.1%)

Class 3 - high physical fatigue, high emotional fatigue, high pain, high depressive symptoms (*n* = 151, 23.5%)

## Discussion

In this study, we identified three classes of the pain-fatigue-depression symptom cluster. The majority of long-term PC survivors had no problems with pain, physical, and emotional fatigue, but had moderate depressive symptoms (class 1). The other two pain-fatigue-depression symptom classes were characterized by having high depressive symptoms and a higher burden of emotional fatigue. The result of our study indicates that different classes of the pain-fatigue-depression symptom cluster exist are in line with previous studies [24, 26].

It is interesting that we observed a strong difference in only one fatigue dimension between class 1 and class 2. Physical fatigue was similarly low in both classes, but emotional fatigue, which had a mean score of 0 in class 1, was higher (mean score of 17.9) in class 2. Moreover, the depressive symptoms mean score in class 2 (38.5) was almost double that of class 1 (21.3), whereas the mean score for pain was equally low in both classes. This is in line with the fact that the only factor which differentiated survivors between class 1 and class 2 was “having a depression”. In the EORTC QLQ-FA12, emotional fatigue is an expression of “lack of motivation” which is likely to partly overlap with items in the MHI-5 questionnaire assessing motivation and possible anhedonia (lack of positive aspects). As different dimensions of fatigue can lead to different clinical outcomes, this highlights the importance of accurate differential diagnosis for effective clinical management of these symptoms [38, 39].

Moreover, PC survivors in class 3 suffered significantly from other specific comorbidities besides depression (e.g. degenerative disc disease and upper gastrointestinal disease) than PC survivors in class 1, whereas no significant odds ratios for these comorbidities were found when comparing PC survivors of class 1 and class 2. As the prevalence of comorbidities and intensity of fatigue are associated [40], this result is underlined by the large difference in the physical fatigue score between class 1 (mean 12.0) and class 3 (mean 53.5). This result regarding comorbidities is noteworthy, especially in view of previous research showing that there is a potential association between fatigue and increased risk of all-cause mortality in male colorectal cancer survivors, in particular in those with comorbid heart disease [41].

Interestingly, two characteristics that differentiated survivors between class 2 and class 3 were doing more than 1.5 h of vigorous physical activities per week and being obese, such that PC survivors of class 2 were more active and had a lower BMI. These results are comparable with Kim et al. [26], who also observed that subgroups differed regarding physical activity status, and with Thong et al. [35], who reported that overweight/obese colorectal cancer survivors were more likely to be classified in the high fatigue group. Therefore, these data show the need for promoting a physically active lifestyle in order to reduce fatigue and depressive symptoms [42].

Being in class 3 was also associated with higher odds of external-beam radiation therapy (even after adjustment for stage and age (data not shown)), potentially due to long-term adverse treatment effects such as lower poorer sexual functioning and urinary or bowel problems. These problems can persist [43] and remain of concern years after treatment has ended [44]. Persistence of these symptoms is associated with treatment regret [44] and perceptions of faecal or urine body odour were associated with depressive symptoms [45]. In our sample, men in class 3 reported more treatment-related symptoms and were more likely to have comorbid depression.

Overall, PC survivors in class 3 reported significantly lower scores for all functioning subscales and higher symptom burden when compared with classes 1 and 2. Similar trends were observed when comparing classes 1 and 2, whereas PC survivors of class 2 reported significantly poorer scores for some functioning and symptom scale scores. These results are not entirely surprising as we have expected that PC survivors with higher burden from multiple symptoms have decreased HRQoL functioning, similar to a previous study on classes of cancer-related fatigue [35]. However, we were surprised by the extent of the mean HRQoL differences as these were much larger than differences in HRQoL by treatment, age, or years since diagnosis in a similar population [18, 19]. This suggests that HRQoL differences may be better explained by classification of long-term PC survivors to a class of pain-fatigue-depression cluster than based on therapy, age, or time since diagnosis. Regarding PC-specific symptoms, our results are in line with the findings of Baden et al. [23] who investigated the prevalence of the pain-fatigue-depression symptom cluster in PC survivors. Their study showed that PC survivors with all three symptoms were more likely to experience physical symptoms such as incontinence, bowel problems, and symptoms related to androgen deprivation treatment than survivors with 0–2 symptoms of this cluster. However, as we also found distinct characteristics associated with each class, we believe interventions should be tailored to each pain-fatigue-depression symptom cluster class as Thong et al. [35] suggest for subtypes of the cancer-related fatigue cluster. For example, PC cancer survivors characterized by low physical activity and/or high BMI could profit from an intervention involving advice on nutrition and physical activity [46]. Moreover, exercise, pharmacological, psychoeducation, and mind body therapies could improve fatigue and depression [47, 48].

Further studies of longitudinal design that include and focus on long-term cancer survivors are needed to replicate our results and to investigate how pain-fatigue-depression symptom cluster classes could potentially change over time. For example, the study by Kim et al. suggested that while subgroup composition of the PNS cluster can remain consistent, patients may switch between subgroups over time, or that more subgroups emerge [26]. In PC survivors, HRQoL has been found to be lower during and shortly after treatment but to improve and stabilize thereafter [49, 50]; therefore, it would be interesting to see whether a similar effect as described by Kim et al. would be found in PC survivors.

This study has several limitations. First, as this was an exploratory study, results should be interpreted with caution and need to be replicated in future studies. Second, we used the 75th percentile score cut-off as there are no established cut-offs for the physical and emotional fatigue dimensions of the EORTC QLQ-FA12, and for the EORTC QLQ-C30. However, our values and cut-off for pain are within the range of published interquartile ranges of the chronically ill patients [51]. For the physical and emotional fatigue dimension of the EORTC QLQ-FA12, no literature for a comparable cohort could be found. Third, due to small sample sizes with respect to specific clinical and sociodemographic characteristics, several confidence intervals (Table 4) are wide, resulting in less statistical power for some comparisons. Finally, as fatigue, pain, and depressive symptoms were only assessed at one time point, we could not identify changes over time.

Nevertheless, this is the first study performed in PC survivors identifying classes of the pain-fatigue-depression cluster. Moreover, a multidimensional fatigue questionnaire was used which allowed for differentiation of fatigue dimensions in the identified classes. Additionally, we could assess the association of a broad range of clinical, demographic, and lifestyle characteristics with the identified classes, and the associations of the classes with HRQoL and PC-specific symptom burden outcomes.

In conclusion, we found three distinct classes of the pain-fatigue-depression cluster. These classes were associated with treatment, comorbidities and lifestyle factors, and HRQoL outcomes. Therefore, improving classification of PC survivors according to severity of multiple symptoms could assist in developing interventions tailored to survivors’ needs to improve HRQoL outcomes.

## Supplementary Information

ESM 1 (DOCX 61 kb)

## Data Availability

The data that support the findings of this study are available on request from the corresponding author. The data are not publicly available due to privacy or ethical restrictions.
