# Supplementary material for: Identifying classes of the pain, fatigue, and depression symptom cluster in long-term prostate cancer survivors—results from the multi-regional Prostate Cancer Survivorship Study in Switzerland (PROCAS)
Source: Support Care Cancer. 2021 Apr 13;29(11):6259–69. doi: 10.1007/s00520-021-06132-w (PMC8464556; doi:10.1007/s00520-021-06132-w)
Supplement: Supplementary file 1 — (DOCX 61 kb) [file 520_2021_6132_MOESM1_ESM.docx]

**Supplementary Material**


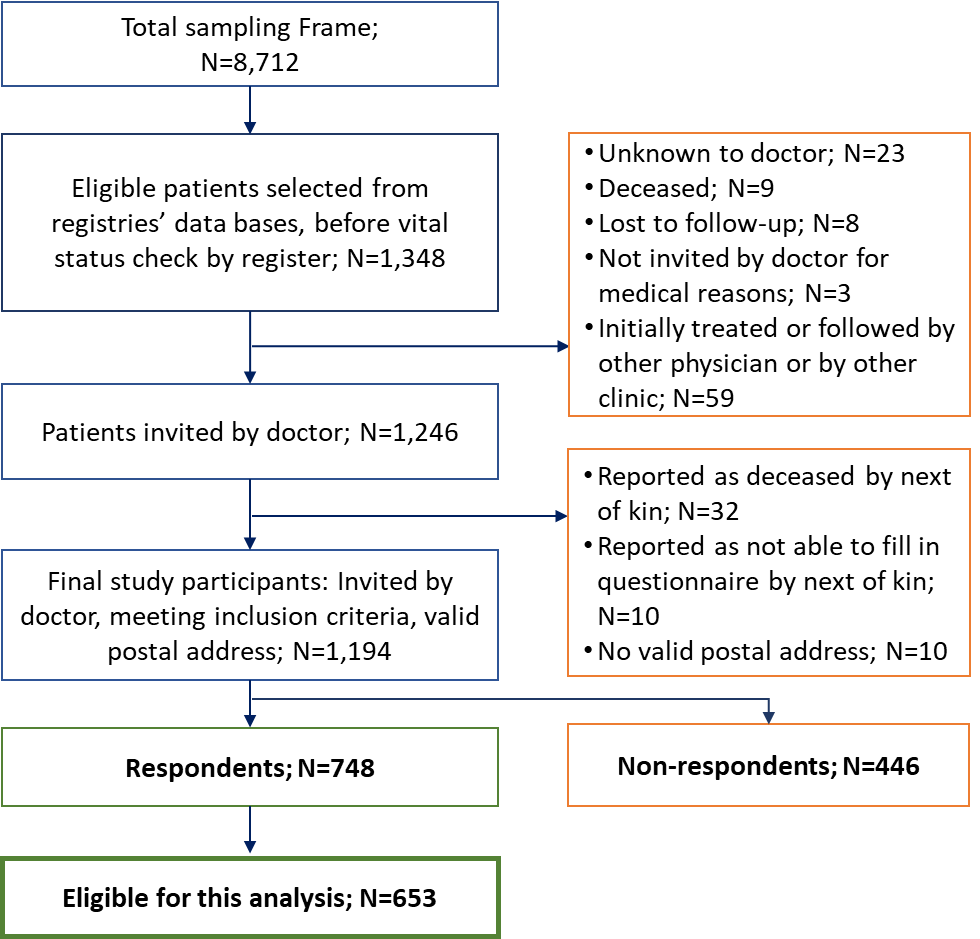


| **Table S1: Mean EORTC QLQ-C30 and EORTC PR-25 scores of PC survivors** | | | |
| --- | --- | --- | --- |
|  | **Mean** | **SE** |  |
| **EORTC QLQ-C30 Functioning scales** |  |  |  |
| **Global health/QoL** | 78.1 | 0.74 |  |
| **Physical functioning** | 90.1 | 0.62 |  |
| **Cognitive functioning** | 85.8 | 0.69 |  |
| **Emotional functioning** | 85.3 | 0.75 |  |
| **Role functioning** | 87.2 | 0.74 |  |
| **Social functioning** | 85.9 | 0.90 |  |
| **EORTC PR-25 scales** |  |  |  |
| **Urinary symptoms** | 18.2 | 0.66 |  |
| **Urinary bother^1^** | 27.9 | 2.32 |  |
| **Bowel symptoms** | 5.7 | 0.43 |  |
| **Hormonal treatment-related symptoms** | 11.5 | 0.48 |  |
| **Sexual activity** | 41.5 | 1.13 |  |
| **Sexual functioning^1^** | 47.2 | 1.05 |  |
| EORTC QLQ-C30: higher scores on functioning scales indicate better functioning or global health.  EORTC QLQ-PR25: higher score in the EORTC QLQ-PR25 represents a greater symptom burden or better sexual functioning and activity.  ^1^Smaller sample sizes and no imputation was performed as the questions referring to these scales are conditional | | |  |

| **Table S2: Correlations between physical fatigue, emotional fatigue, pain and depressive symptoms scores and** **EORTC QLQ-C30 and EORTC-PR25 scales. Correlation coefficients are shown.** | | | | |
| --- | --- | --- | --- | --- |
|  | **physical fatigue** | **emotional fatigue** | **pain** | **depressive symptoms** |
| **EORTC QLQ-C30 Functioning scales** | | | | |
| **Global health/QoL** | **-0.46** | **-0.41** | **-0.42** | **-0.35** |
| **Physical functioning** | **-0.48** | **-0.32** | **-0.45** | **-0.27** |
| **Cognitive functioning** | **-0.41** | **-0.36** | **-0.29** | **-0.36** |
| **Emotional functioning** | **-0.48** | **-0.47** | **-0.34** | **-0.51** |
| **Role functioning** | **-0.44** | **-0.36** | **-0.50** | **-0.28** |
| **Social functioning** | **-0.37** | **-0.36** | **-0.35** | **-0.29** |
| **EORTC PR-25 scales** | | | | |
| **Urinary symptoms** | **0.35** | **0.29** | **0.27** | **0.25** |
| **Urinary bother^1^** | **0.19** | **0.16** | **0.10** | **0.17** |
| **Bowel symptoms** | **0.33** | **0.29** | **0.21** | **0.33** |
| **Hormonal treatment-related symptoms** | **0.40** | **0.39** | **0.22** | **0.32** |
| **Sexual activity** | **-0.10** | **-0.07** | **-0.13** | **-0.10** |
| **Sexual functioning^1^** | **-0.15** | **-0.19** | **-0.12** | **-0.07** |
| ^1^Smaller sample sizes and no imputation was performed as the questions referring to these scales are conditional | | | | |

| **Table S3: Minimum value, 25th interquartile score, median, 75th interquartile score and maximum value of physical fatigue, emotional fatigue, pain and depressive symptom*, by class of pain-fatigue-depression cluster** | | | | | |
| --- | --- | --- | --- | --- | --- |
|  | Minimum Value | ^25th^ interquartile score | Median | 75^th^ interquartile score | Maximum Value |
| **Class 1** |  |  |  |  |  |
| **Physical Fatigue** | **0.0** | **0.0** | **6.7** | **20.0** | **66.7** |
| **Emotional Fatigue** | **0.0** | **0.0** | **0.0** | **0.0** | **0.0** |
| **Pain** | **0.0** | **0.0** | **0.0** | **16.7** | **100.0** |
| **Depressive Symptoms *** | **1.0** | **15.0** | **20.0** | **25.0** | **43.8** |
| **Class 2** | | | | |  |
| **Physical Fatigue** | **0.0** | **6.7** | **13.3** | **20.0** | **26.7** |
| **Emotional Fatigue** | **0.0** | **11.1** | **11.1** | **22.2** | **66.7** |
| **Pain** | **0.0** | **0.0** | **0.0** | **16.7** | **100.0** |
| **Depressive Symptoms*** | **10.0** | **30.0** | **35.0** | **50.0** | **70.0** |
| **Class 3** |  |  |  |  |  |
| **Physical Fatigue** | **33.4** | **40.0** | **43.3** | **66.7** | **100.0** |
| **Emotional Fatigue** | **0.0** | **11.1** | **33.3** | **55.6** | **100.0** |
| **Pain** | **0.0** | **0.0** | **33.3** | **50.0** | **100.0** |
| **Depressive Symptoms*** | **15.0** | **35.0** | **45.0** | **55.0** | **90.0** |
| * Score is the reverse of the depressive symptom score for better comparability to the other scores | | | | | |
